# Medical Society of County of New York

**Published:** 1869-10

**Authors:** Geo. T. Elliot

**Affiliations:** President, in the Chair


### MEDICAL SOCIETY OF COUNTY OF NEW YORK.

*Stated Meeting, June* 7, 1869.

Dr. GEO. T. ELLIOT, Jr., President, in the Chair.

The President announced the election of Dr Robert McNeil to membership.

Dr. Salvatore Caro read a paper upon *the Treatment of Summer Complaints by the Bromide of Potassium.**

Although the title of this paper comprises the entire range of diarrhœal diseases both of adult and infantile life, special reference was had to the diarrhœal diseases of infancy, cholera-infantum standing prominent in the list. The causes, symptoms, progress, and termination of this complaint were described at length, as well as, in a general way, the treatment which is usually considered orthodox in these cases. Then stating that an accidental success in the treatment of several cases, wherein he had administered the bromide for other purposes than to control the diarrhœa, had led him to more fully test the value of this remedy, the speaker narrated twenty cases which might be taken as types of the cases usually met with in practice, and which were selected from 163 recorded cases occurring under his own observation. We present herewith, from the *Record*, five of the cases in full:

III. John Sinott, 28 months old. I took charge of him on

* This paper is published in full in the *Medical Record* of July 1, 1869.

the 1st of August. For several days he had been suffering from vomiting and purulent discharges from the bowels. I prescribed aromatic syrup of rhubarb with laudanum, but without effect. On the 2d, I gave twenty drops every hour, of a mixture of ten grains of bromide of potassium, in an ounce of mucilage, with twenty minims of krameria. After a few doses the child slept, and upon awakening asked for bread and butter. Vomiting ceased. The flux of the bowels changed from purulent to yellow; and the 24 to 30 passages, every 24 hours, diminished to six. He became convalescent, and on the 8th was discharged.

IV. J. A. Criger, 20 months old; has twelve teeth. According to his mother's statement, he has never given trouble, eating and drinking whatever was offered him. On the 3d of August he fell from a chair, striking his buttock, apparently receiving no injury. During the night his bowels became loose, accompanied by a dirty-water-like substance. In the morning he had convulsions. During the afternoon I found him very comatose; pulse from 95 to 100; pupils dilated; hemiplegia of the right side; alvine discharges, from twelve to fourteen, every 24 hours, of a thin fetid matter. About every six hours, convulsions of ten minutes' duration, accompanied by vomiting. I prescribed revulsives to the lower extremities and arms; and, every two hours, twenty drops of a mixture of one drachm of bromide of potassium, in an ounce of mucilage. The motion of the bowels and vomiting ceased.

After a few days, as the comatose and hemiplegic condition continued, iodide of potassium brought matters right. On the 1st of November he had another fall, merely affecting his bowels by free motion; the bromide of potassium immediately stopped it.

V. John Balheimer, 13 months old; has six teeth; is fed by the bottle. On the 15th of August I saw the child for the first time. He had been suffering for 14 days from inflammatory dysentery, having passages of a purulent and bloody nature, from 20 to 24, every 24 hours. His thirst was intense; he vomited every fluid offered him. The eyes were sunken, pupils dilated, skin corrugated and spotted blue, body cold, tongue red and dry, pulse imperceptible; no urine. In addition to this, he had bronchitis, with severe cough. I prescribed 1 scruple of bromide of potassium, in an ounce of mucilage, with ½ drachm of tincture of krameria, 20 drops every two hours. The passages decreasd. On the 16th day of his sickness, and the second under my care, he was able to eat and sleep. His passages were only 4 in 24 hours, and of a healthy appearance.

In order to allay the bronchial cough, I prescribed 10 drops of fluid extract of poppy, to be taken every two hours. On the 1st of September, he was discharged. A few days later he had a relapse, and on account of distance was placed under the care of a homœopathist, and died.

On the 15th, an elder brother was brought to me suffering in a similar manner from dysentery. The disease was immediately checked by the bromide of potassium.

X. J. McEvoy, 12½ years old: a healthy boy, attending school. August 13th, having eaten a bellyful of green apples, he was seized with cramps in the belly and legs, colic pains, vomiting, and loose bowels. For 48 hours his parents doctored him with castor-oil, paregoric, cholera drops, mustard baths, etc., etc., but without giving relief. I was sent for on the 15th, the third day of his sickness. I found him with eyes sunken, skin cold and corrugated, extremeties bluish, which upon being touched would leave the white impress of the fingers for more than 25 seconds. The abdomen was almost struck to the vertebral column; breath hurried and hot; tongue pale; voice scarcely audible. The boy was shivering with cold, although covered with mustard plasters, blankets, etc., and in an exceedingly hot, low-ceilinged room on the top floor. His thirst was intense. The drinks given him were, rice-water, beef-tea, brandy-and-water, ice-water, milk-whey, etc. No sooner taken than ejected indiscriminately, both up and down, almost in their natural state. I thought this case almost hopeless, but, trusting to the efficacy of the bromide of potassium, I prescribed ½ drachm in an ounce of water a teaspoonful every two hours; also a flaxseed meal poultice over the abdomen. The second dose caused great reaction, stopping the vomiting and flux of the bowels, and the boy fell asleep. The next morning I found him in his hot unhealthy room, laughing and well.

I have had 25 similar cases, ranging from 12 to 46 years of age.

XIV. J. Higgins, a healthy infant, deprived of the breast from his mother's dying seven days after confinement. When only three days old he began to suffer from retention or meconium. All nourishment was ejected with great force, even the breast milk of a woman confined five days before his birth. The stomach, from the irritating action of the meconium, could not retain either medicine or food, nor could the bowels be moved. Ten grains of the bromide of potassium in an ounce of orange-flower water, ten drops every hour, put a stop to all disagreeable symptoms; the baby slept, its bowels moved, and, after the discharge of the meconium, it commenced to nourish from the bottle without any further disagreeable results.

I had three similar cases, not owing to the mothers' death, but the infants being deprived of the maternal nourishment from other causes.

The paper concluded as follows:

Now let us speak on the merits of the drug. Taking for granted that, in matters of fact, in medicine particularly, we can judge better *a posteriori* than *a priori*, I submit my cases and conclusions to this enlightened body. Not accepting the doctrine of some of the modern writers, that the intestinal flux is a symptom of some inflammatory affection, but adopting the opinion of the celebrated Capuron, that infancy being naturally the age of nervous sensitiveness, susceptible to internal and external impressions, easily irritated or calmed, and judging from several other cases besides those just read, I think that, in its beginning, summer-complaint arises from an over-excitement of the nervous and vascular systems, and that therefore the bromide of potassium affects it, and acts as a sure cure.

I cannot better explain the action of the bromide of potassium on the system, than by quoting the words of Headland, found on page 272 of his work, "*On the Action of Medicines*," "The bromide of potassium, a medicine of the mineral kingdom, has been much recommended lately for its power of producing sleep; it is, however not a true soporific, but rather a general sedative. As far as my experience goes, it acts by allaying irritability of the brain, spinal cord, and sexual system, and thus may indirectly cause sleep." And on page 283,—"The bromide of potassium, a remedy of recent introduction, is exceptional among nerve medicines as, belonging to the mineral instead of the vegetable kingdom. It quiets the nervous system generally, allays pain, promotes sleep, and subdues a morbid irritability of cutaneous or mucous surfaces, by its influence over functional disorders of the nervous centres. It is only indirectly soporific, as far at least as I have been able to judge." Mr. Gubler considers the bromide of potassium a general sedative to the nervous system, allaying irritability of the mucous membranes, and at the fauces and genital passages especially. He thinks it hypnotic, causing sleep by its sedative action on the whole nervous system. M. Vigouroux thinks it acts by diminishing vascularity of the great nervous centre. It is therefore the remedy, *par excellence*, for the nervous complaints that are common in large cities.—(*L'Union Medicale*, 1864.)

I have never discovered any unpleasant effects produced by the use of the bromide of potassium, and have always found it to answer my purposes. If locally applied, it reduces the excessive heat found in the mouth of the baby, caused by the inflamed condition of the gums. On introducing the thermometer into the mouth or anus of the baby, I have seen the temperature fall four or five degrees. The drug is an anæsthetic to the nerves of the mucous membrane of the alimentary canal from the mouth to the rectum, of the urethra, the conjunctiva, and the nares. It is also a diuretic. In cases where the urine has not been voided for 24 hours, after the first dose of the bromide it is freely passed; the skin commences to feel warm, and becomes covered with a gentle perspiration, removing uræmic symptoms, causing general reaction, and invariably the decrease of the intestinal flux, and an absolute cessation of vomiting.

The medicine being almost tasteless, and of no bulk, it is easily taken by the most fastidious and troublesome child. I generally prescribe from 10 to 30 grains in an ounce of vehicle, either mucilage or orange-flower water, for their pleasant taste; the dose being 10 to 30 drops every hour or two, varying according to the age of the patient, and the acuteness of the case. I seldom use astringents with it; but, if required, I select the tincture of krameria, as less disgusting than kino and others. For local application, I mix the salt with mel rosarum, generally using one scruple of the first to an ounce of the latter, permitting the mother to rub it on the gums with her finger *ad libitum*. When used in large doses, I have not found very satisfactory results from the bromide of potassium; but I always succeeded with minute doses.

You will observe that, of the cases I have just reported, only three died, and the fatal result in these was due not to the bowel complaint, but to other causes. I had also four cases which I purposely and obstinately treated according to the generally-adopted system. To my regret I must confess, that but one, after a severe and hard struggle, recovered; the other three died.

Dr. Calkins deemed hot-water and hot-vapor baths invaluable auxiliaries in the treatment of the bowel complaints of children, and related a fatal case, which he thought had suffered from their omission.

Dr. Garrish had found the bromide fail to control the nausea and vomiting of pregnancy, and asked Dr. Caro's experience.

Dr. Caro had not used the drug in pregnancy, as he had gained the impression, from something in his reading, that it was emmenagogue, and might do harm. He had found it immediately arrest the vomiting in the case of a woman with Bright's disease, who had, for three or four weeks, been unable to retain food. It had stopped the nausea and vomiting in a case of typhoid double pneumonia.

Dr. Chadsey had been using the bromide quite freely, for the last three months, in affections of the stomach and bowels. It had happily relieved the only case of cholera-infantum he had chanced to have in at that time. In a case of vomiting, in the commencement of pregnancy, which nothing else would check, it had been promptly effective, and no bad results had followed—indeed, none had been anticipated. A druggist, having a very large prescription business, had said that the consumption of the bromide had quadrupled within a short time, and that it was now greater than that of the iodide, which it was replacing in many instances.

The President was asked whether he considered the bromide an emmenagogue. He replied that he had used it in many cases of pregnancy, as well to control the nausea and vomiting as for other indications, and his experience was that it had no emmenagogue action whatever. He was in the habit of employing it freely, and in large doses, in dysmenorrhœa, to gain its sedative effect, and he had never seen it increase the flow. He could not, therefore, think we were justified in refraining from its use on account of the pregnant condition. He had not found it so satisfactory a remedy in the nausea and vomiting of pregnancy as to lead him to give it the first rank; but neither did he know any other remedy to which this could be assigned. In the treatment of such a symptomatic disorder we must expect failure. It was trying to remove the effects of the thorn in the flesh, without removing the thorn. Among the articles that had lately appeared, commending the bromide in the various forms of nausea, was one by Dr. Storer, of Bos ton, showing that it counteracted the nauseating effects of ether.

With regard to the class of cases related by Dr. Caro, Dr. Elliot yielded entirely to him in experience, and could speak, from personal knowledge, of his accuracy of observation and record. His testimony was of such value that it would probably more or less shape the practice of his hearers this summer. In the case of a rachitic little child, suffering from whooping-cough and bronchial catarrh, with a good deal of diarrhœa, refusing nourishment, and lying on the bed, with hot skin, restless and fretful, Dr. E. had given the bromide alone, and its effect was delightful. It had produced like results in one or two other cases equally unpromising.

As to the extended use of the salt, Dr. Chadsey was certainly right. Probably every druggist was now selling much more than four times as much of it as a few years ago. For his own part, while prescribing it much more frequently than before, it was not so lately that Dr. E. had been awakened to its value. Fourteen years ago it was his favorite remedy in spasmodic croup, and bronchial catarrh, with irritation of the respiratory passages. Having about that time a troublesome case of spasmodic croup, he had at last given the mother the prescription, labelled "Preventive for croup," to be used whenever she saw an attack coming on. On her return from Europe, the lady said it had worked so well that she had given the prescription to some sixty families.

The hypnotic action of the bromide was so well known as to need no comment. The danger was, now, that too many of the public would acquire the habit of taking it, as a regular thing, to procure a quiet night's sleep. Dr. E. believed that a gentleman of this city had lost his life from its excessive and habitual use. A prescription given to secure sleep, this man had kept and had renewed, again, and again, until he used to lie half the day in a state of languor. Coming under the speaker's care, his habit was discovered, and finally given up; but not before it had produced the prostration which was probably the cause of his death.

Dr. Caro, in response to questions, said that he had commonly given the small doses—¼ of a grain to 3 grains—mentioned in his record of cases; and that he had never, save in a single instance, given a dose larger than 8 grains. He had seen no eruption follow, except in one case, and there he thought it not attributable to the bromide.

Dr. Kennedy thought the dose of the drug might be considered very indefinite. He had himself taken a large amount of it last winter, for an obscure trouble in the head and pain in the shoulders—the doctors were undecided about the diagnosis, but agreed in recommending the bromide. He had begun with a solution of 2 drachms of the salt to 4 ounces of water, a teaspoonful every three or four hours. This dose he increased till he was taking 2 and 4 drachms of the salt daily. Then he bought ½ a pound of it and made a saturated solution, of which he took at first a teaspoonful, and afterward a tablespoonful, four times a day; and he might have taken more without injury. It gave a very pleasant night's rest, and a delightful feeling of languor and lassitude, which was continued for days and weeks. He had observed the eruption; also that the medicine disturbed the bowels and stimulated the kidneys; but, in his own case, it did not at all disturb the stomach, simply producing an agreeable sense of warmth. He believed that, where the nervous system is irritated, from whatever cause, and it becomes necessary to soothe either body or mind, the bromide is an excellent remedy. He would recommend it to be taken freely in such cases, and especially by rum-drinkers.

The President stated that a saturated solution in water was one part to four.

Dr. Farnham referred to the cases reported by Dr. Hammond, where the bromide, in large doses, had produced insanity.

The President had once given ½ an ounce in the course of one night, in a bad case of delirium tremens. The morning came, and it had produced no perceptible effect; but a shower-bath on the head and neck put the patient to sleep in a couple of hours. He would hardly give such a dose again; for, although some persons might bear it well, there was undoubted testimony to its evil effects on others. We should remember the fate of Fountain, who to prove his views of the harmlessness of chlorate of potassa, took the dose which killed him.

Dr. Howard had used 10 grain doses, every two hours, with great benefit, in cases of sick headache.—*N. Y. Med. Jour.*

